# Economic Disparities in Palliative Care Utilization Among Cancer Patients in Saudi Arabia: A Socioeconomic Stratification Analysis

**DOI:** 10.3390/curroncol33040218

**Published:** 2026-04-15

**Authors:** Thurayya Eid, Norah M. Alyahya, Abdulaziz M. Alodhailah, Bader M. Almutairy, Faihan F. Alshaibany, Waleed M. Alshehri

**Affiliations:** 1 Department of Medical-Surgical Nursing, College of Nursing, King Saud University, Riyadh 11451, Saudi Arabia; teid@ksu.edu.sa (T.E.);; 2 Department of Community and Psychiatric Mental Health Nursing, College of Nursing, King Saud University, Riyadh 11451, Saudi Arabia; nora@ksu.edu.sa (N.M.A.);; 3 Department of Nursing Administration and Education, College of Nursing, King Saud University, Riyadh 11451, Saudi Arabia

**Keywords:** palliative care, health disparities, socioeconomic status, income inequality, cancer, healthcare access, health equity, Saudi Arabia

## Abstract

Economic inequities in healthcare access persist globally, yet the impact of income on palliative care (PC) utilization in Middle Eastern contexts remains empirically understudied. This cross-sectional study of 200 cancer patients in Riyadh, Saudi Arabia, employed a socioeconomic stratification analysis to examine income-stratified differences in PC awareness and access. Using chi-square and linear-by-linear association tests, results revealed pronounced income gradients; awareness increased from 41.9% in the low-income group to 71.9% in the high-income group (*p* = 0.001), demonstrating a significant dose–response trend. Access disparities were even more striking, with only 35.5% of low-income patients utilizing services compared to 76.1% of high-income patients (*p* < 0.001), representing a 40.6 percentage-point gap. After multivariable adjustment, after controlling for age, gender, education, and geographic living region, the results of logistic regression analysis showed that cancer patients with high income were more than three times as likely to access PC services compared with lower-income cancer patients (OR = 3.32; 95% CI: 1.83–6.02; *p* < 0.001). Geographic stratification further indicated that income disparities were significantly amplified in peripheral regions compared to the Central region (*p* = 0.072 for interaction), where service scarcity exacerbates economic barriers. These findings underscore that economic barriers operate through awareness gaps and structural obstacles like transportation and opportunity costs. Addressing these inequities requires multifaceted strategies, including financial support and geographic service expansion, to ensure equitable PC distribution under the Vision 2030 framework.

## Simple Summary

This study was conducted to investigate how income levels affect access to palliative care for cancer patients in Saudi Arabia. By analyzing data from 200 patients, the research identified a stark economic divide, where high-income patients were three times more likely to access these services compared to those with low incomes. Major barriers faced by the lower economic group included transportation costs, lost wages due to time off work, and additional out-of-pocket medical expenses. This gap was found to be significantly worse in remote areas where healthcare facilities are limited. These findings emphasize the need for new policies, such as transportation subsidies and the expansion of services to rural regions, to ensure that palliative care is accessible to all members of society regardless of their financial status.

## 1. Introduction

Economic disparities represent one of the most persistent, pernicious, and ethically troubling sources of healthcare inequity globally [1]. Individuals with lower socioeconomic status consistently experience demonstrably reduced access to high-quality healthcare services, delayed diagnosis, and inferior treatment quality across diverse diseases and care settings [2]. These systematic disparities violate fundamental ethical principles of healthcare justice and directly undermine a health system’s capacity to serve all populations effectively [3]. In the field of oncology, the impact of financial instability is particularly devastating, as cancer patients with limited resources often face a constellation of compounding barriers [4,5]. These include transportation hurdles to specialized facilities located in urban centers, significant opportunity costs from lost wages, and out-of-pocket expenses for medications not fully covered by national insurance [5,6]. Such economic burdens create a destructive cycle of disadvantage, where financial strain accelerates health deterioration and reduces the quality of life during critical illness phases [5,6].

In the global context of palliative care (PC) services, significant inequities exist across healthcare systems. According to the 2024 Global Atlas of Palliative Care, Saudi Arabia ranks 47th globally and 3rd within the Middle East and North Africa region in terms of PC service development and accessibility [7]. The Saudi Arabian PC system encompasses multiple service delivery models, including specialized hospice units within tertiary hospitals, outpatient palliative care clinics integrated within oncology departments, community-based home care programs, and consultation services available in both public and private healthcare facilities. These services, while expanding under the Vision 2030 initiative, remain concentrated primarily in urban centers, particularly Riyadh, leaving peripheral and rural regions substantially underserved [8].

In the context of Saudi Arabia, the healthcare landscape is undergoing a profound transformation. Under the Vision 2030 framework, the Kingdom has made a comprehensive commitment to enhancing healthcare quality, efficiency, and equity [8,9]. While the Ministry of Health provides substantial public coverage that establishes a foundation different from fully privatized systems, significant variations in service availability persist across income levels and geographic regions [10]. Palliative care (PC) services have expanded substantially in tertiary urban centers; however, the specific impact of income on PC utilization remains empirically understudied [11,12]. Without a systematic understanding of these economic barriers, expansion efforts risk further entrenching disparities by concentrating services in areas primarily accessible to wealthier populations, thereby leaving the most vulnerable patients behind [13,14].

The concentration of specialized PC services in urban hubs creates intersecting economic and geographic disadvantages [15]. Lower-income patients in peripheral regions face double jeopardy: greater travel distances coupled with fewer financial resources to overcome transportation and accommodation costs [16]. This intersection of disadvantage necessitates a sophisticated approach to service distribution and patient support [17]. Nurses, occupying strategic frontline positions in oncology, are uniquely capable of addressing these inequities [18]. Through systematic assessment of a patient’s economic circumstances and providing navigation assistance to social support programs, nurses can function as essential equalizers in the healthcare system [19,20]. Understanding the magnitude and distribution of these economic disparities is therefore essential for targeting nursing interventions and advocating for systemic changes [21,22].

This study is grounded in a health equity framework that conceptualizes access as a result of multiple intersecting factors, including structural costs, systemic referral patterns, and geographic concentration. By treating economic status as a fundamental cause of inequity, we move beyond viewing poverty as a monolithic disadvantage and instead examine the specific pathways through which it affects care [23,24]. Consequently, this study aimed to examine economic disparities in PC awareness and access among cancer patients in Saudi Arabia. We focused on the independent effect of income as a predictor of access, the existence of linear dose–response trends across income strata, and how geographic location modifies these economic effects. We hypothesized that substantial income gradients would characterize both awareness and access and that income would remain a significant predictor even after controlling for other sociodemographic factors. The findings carry direct implications for healthcare policy and the development of equitable PC systems.

## 2. Materials and Methods

### 2.1. Study Design and Setting

This investigation employed a descriptive cross-sectional survey design to examine the associations between income levels, palliative care (PC) awareness, and service access among cancer patients. This observational approach allowed for an efficient examination of current utilization patterns while providing a foundational basis for generating hypotheses for future intervention research. The research was conducted across multiple healthcare settings in Riyadh, Saudi Arabia, including three tertiary oncology centers providing advanced cancer treatment and consultative services, two regional hospitals offering oncology and general PC services, and two community-based cancer patient support organizations.

This diversity of settings ensured representation of cancer patients from varied socioeconomic backgrounds, educational levels, and degrees of prior exposure to PC services. Diverse recruitment venues reduce selection bias inherent in single-site recruitment.

### 2.2. Population and Sampling

Inclusion Criteria: Adult cancer patients (≥18 years of age) actively receiving oncology treatment or follow-up care were eligible. All participants were eligible for palliative care services according to national oncology guidelines, encompassing patients with any cancer stage or disease trajectory. Participants had received or were eligible to receive various levels of PC services, ranging from symptom management and advance care planning to comprehensive supportive and end-of-life care. Additional criteria included: confirmed cancer diagnosis of any site, Saudi Arabian residency, and demonstrated capacity to provide informed consent.

Exclusion Criteria: Patients with severe cognitive impairment precluding comprehension of questionnaires and patients with illness severity deemed by treating clinicians to create undue burden were excluded.

Recruitment: A stratified purposeful sampling approach was employed to recruit the 200 cancer patients participating in this study. The sampling strategy explicitly prioritized economic stratification to ensure adequate representation of patients across diverse income levels. Recruitment was conducted systematically across the seven participating healthcare settings: three tertiary oncology centers, two regional hospitals, and two community-based cancer patient support organizations. From the three tertiary centers, 85 patients were recruited; from the two regional hospitals, 68 patients were recruited; and from the two community-based organizations, 47 patients were recruited. Within each setting, research staff identified eligible patients sequentially from appointment schedules or organizational records and approached potential participants to assess eligibility and obtain informed consent. This systematic approach, combined with explicit economic stratification, reduced selection bias compared to unstandardized convenience sampling while maintaining feasibility in clinical settings.

### 2.3. Income Stratification

Participants were explicitly stratified into three income groups based on their reported monthly household income in Saudi Riyals (SAR), consisting of a low-income group earning less than 5000 SAR per month (*n* = 62; 31.0%), a middle-income group earning between 5001 and 10,000 SAR per month (*n* = 71; 35.5%), and a high-income group earning more than 10,001 SAR per month (*n* = 67; 33.5%). These income categories reflect meaningful distinctions in purchasing power, healthcare access capacity, and financial vulnerability within the Saudi Arabian context, based on contemporary national income distribution data from the General Authority for Statistics. Furthermore, these categories approximate tertiles within the sample, which specifically enables the rigorous examination of dose–response patterns across the socioeconomic spectrum.

### 2.4. Data Collection Instruments

Sociodemographic and Clinical Information Form: This investigator-developed instrument comprehensively captured demographic data including age (continuous), gender, marital status, educational attainment (primary/secondary/university), monthly household income (continuous with categorical groupings), insurance status (Government, Private, or No Insurance), and geographic region of residence (Central, Eastern, Western). Clinical variables included cancer diagnosis site, disease stage (localized/regional/metastatic), and treatment status (active treatment/remission/palliative-focused care).

The Palliative Care Awareness and Accessibility Scale (PCAAS): This 12-item instrument was adapted from the Palliative Care Attitudes Scale (PCAS-9) [25]. The original PCAS-9 comprises 9 items organized into three subscales: emotional, cognitive, and behavioral dimensions of attitudes toward palliative care. For this study, a 12-item adapted version was developed to specifically measure two distinct dimensions, awareness and accessibility, which were not comprehensively assessed by the original 9-item instrument. The adapted PCAAS was created following a rigorous development process that included content review by five palliative care specialists, who identified items most relevant to the awareness and accessibility constructs specific to the Saudi Arabian context. The awareness dimension comprises 7 items assessing knowledge of PC’s purpose, its integration with cancer treatment, its benefits for quality of life, and its overall availability. The accessibility dimension comprises 5 items evaluating the perceived feasibility of accessing services, including participants’ awareness of specific referral pathways.

Items employ 5-point Likert scaling (1 = strongly disagree; 5 = strongly agree). For primary analyses, awareness and accessibility responses were dichotomized at the median score to create binary outcome variables suitable for logistic regression modeling. The instrument demonstrated acceptable internal consistency (Cronbach’s α = 0.84) in this sample. In the pilot sample of 20 cancer patients, the instrument demonstrated a Cronbach’s alpha coefficient of 0.81, indicating good internal consistency with the adapted version. The range of instrument scores was 12–60, with higher scores indicating greater awareness and accessibility. Instrument development included rigorous translation procedures: forward translation to Arabic, back-translation to English ensuring semantic equivalence, expert review by PC specialists, and pilot testing among 20 cancer patients verifying comprehensibility and cultural appropriateness.

### 2.5. Data Collection Procedures

Following institutional ethical approval and informed consent procedures, participants completed questionnaires in their preferred format: paper-based surveys during outpatient clinic visits or electronically via secure REDCap platforms for online recruitment. A total of 8 trained oncology nurses with minimum 2 years of experience in oncology and formal training in palliative care and research ethics explained study objectives, assessed eligibility, facilitated informed consent, and distributed questionnaires. Nurses remained available to clarify questionnaire items and provide psychological support if participants experienced distress. Average questionnaire completion time was 10–15 min.

### 2.6. Statistical Analysis

Data analysis was conducted in IBM SPSS Statistics version 26 (IBM Corp, Armonk, NY, USA) following a structured analytical strategy:

Descriptive Statistics: Frequency distributions, percentages, means, and standard deviations characterized the sample by income group, identifying sociodemographic patterns across income strata.

Bivariate Associations: Chi-square tests examined associations between categorical income strata and binary outcomes (awareness high/low; access yes/no). These tests assessed whether income–outcome associations achieved statistical significance.

Dose–Response Relationships: Linear-by-linear association tests specifically examined whether awareness and access demonstrated dose–response trends, progressively increasing with income level, rather than simply differing between groups. Significant dose–response relationships strengthen the inference that income itself drives disparities rather than arbitrary categorization artifacts.

Multivariable logistic regression was employed to examine income as an independent predictor of palliative care access while simultaneously controlling for several potentially confounding variables. These covariates included age, which was analyzed both as a continuous variable and as a categorical variable (≥51 years) for the regression model, and gender, comparing female versus male participants. Additionally, the model accounted for educational attainment by comparing university-educated individuals with those holding a secondary education or less, as well as geographic region, contrasting residents of the Central region with those in peripheral areas. This comprehensive model did not include insurance status as a covariate despite its conceptual importance, as preliminary bivariate analysis revealed high multicollinearity with income (variance inflation factor = 5.2), making simultaneous inclusion problematic for effect estimation. Insurance status as a potential effect modifier and its relationship to income are discussed in the Limitations Section. This rigorous approach allowed for the isolation of the specific effect of income on service utilization independent of other sociodemographic factors.

This approach isolates income’s effects from confounding by correlated sociodemographic factors. Prior to conducting the multivariable logistic regression analysis, assumptions were systematically evaluated. Multicollinearity was assessed using variance inflation factors (VIF), with all VIF values < 3.0 except that for the income–education relationship (VIF = 4.8), indicating acceptable collinearity [26,27]. Model fit was evaluated using the Hosmer–Lemeshow goodness-of-fit test, which confirmed adequate model calibration (χ^2^ = 4.32, *p* = 0.368). Receiver operating characteristic (ROC) curve analysis demonstrated good discriminative ability with an area under the curve (AUC) = 0.76 [28,29]. Results are reported as odds ratios (OR) with 95% confidence intervals (CI). Statistical significance was set at *p* < 0.05 (two-tailed).

Stratified Analyses: To examine whether income effects varied by geographic region, stratified analyses compared income gradients in Central versus peripheral (Eastern and Western combined) regions. Interaction terms (income × region) tested whether geographic region statistically modified income’s effects.

Awareness Logistic Regression Analysis: In response to reviewer recommendations, an additional logistic regression analysis was conducted to examine income as a predictor of palliative care awareness, using the same model structure and covariates employed for the access analysis. This analysis provides parallel assessment of awareness determinants and enables comparison of income effects across the two primary outcomes of interest.

### 2.7. Ethical Considerations

Ethical approval for this study was obtained from the Institutional Review Board of King Saud University (Approval #KSU-HE-24960) prior to the commencement of any data collection. All participants provided written informed consent, which was presented in Arabic to ensure full comprehension of the study’s objectives. The consent procedures were designed to guarantee voluntary participation without any form of coercion, providing a clear explanation of how the data would be utilized and the specific privacy protections in place. To maintain the highest standards of anonymity and confidentiality, personal identifiers were strictly removed during the data entry process. Furthermore, all data were stored using secure encryption on password-protected servers with restricted access, and the final analysis was conducted on de-identified datasets to ensure there was no linkage to personal information.

Oncology nurses trained in PC and psychological support remained available to provide assistance for participants experiencing distress during questionnaire completion. Research staff maintained sensitivity to the vulnerability of cancer patients and their families confronting serious illness.

## 3. Results

### 3.1. Sample Characteristics and Income Stratification

The sample comprised 200 cancer patients stratified into three income groups. Table 1 presents the sociodemographic characteristics of participants by income level, including palliative care (PC) service utilization.

A significant age gradient was observed across income categories, with mean age increasing from the low-income group (M = 43.9, SD = 9.8) to the middle-income (M = 48.1, SD = 9.4) and high-income groups (M = 52.8, SD = 8.7) (F = 15.34, *p* < 0.001).

Gender distribution did not differ significantly across income groups, with females comprising 53.2% of the low-income group, 53.5% of the middle-income group, and 44.8% of the high-income group (χ^2^ = 2.18, *p* = 0.337).

Educational attainment was significantly associated with income level. The proportion of participants with university education increased from 25.8% in the low-income group to 46.5% in the middle-income group and 73.1% in the high-income group (χ^2^ = 41.87, *p* < 0.001).

Geographic distribution also varied significantly by income. Participants in the low-income group were more likely to reside in peripheral regions (64.5%) compared with those in the high-income group (43.3%) (χ^2^ = 8.23, *p* = 0.016).

Insurance status differed significantly across income groups. Government insurance was more common among low-income participants (61.3%), whereas private insurance was more prevalent among high-income participants (67.2%) (χ^2^ = 58.45, *p* < 0.001).

PC service utilization showed a significant association with income level. The proportion of participants who had accessed PC services increased from 35.5% in the low-income group to 56.3% in the middle-income group and 76.1% in the high-income group (*p* < 0.001). This variable was included as a covariate in subsequent analyses.

### 3.2. Income Gradients in Palliative Care Awareness

Table 2 presents PC awareness rates stratified by income level. A clear, consistent income gradient emerged: only 41.9% of low-income patients reported high PC awareness compared with 54.9% of middle-income and 71.9% of high-income patients. This 30-percentage-point difference between the highest- and lowest-income groups demonstrates substantial awareness disparities.

Chi-square analysis confirmed these differences achieved statistical significance (χ^2^ = 17.89, *p* = 0.001; Cramér’s V = 0.30). The linear-by-linear association test specifically examining dose–response trends confirmed a highly significant progressive increase in awareness with increasing income (*p* < 0.001). This dose–response pattern strengthens the inference that income itself drives awareness differences rather than reflecting unmeasured confounding.

Interpretation: The substantial income gradient in awareness suggests that higher-income patients possess greater knowledge of PC availability and benefits. This may reflect multiple mechanisms: superior access to health information through internet resources and private healthcare consultations, greater health literacy, and social networks with higher average health knowledge. The gradient indicates that awareness-building initiatives must specifically target lower-income populations currently underrepresented in knowledge distribution.

### 3.3. Income Gradients in Palliative Care Access

Table 3 presents PC access rates by income stratum, revealing pronounced economic disparities in actual service utilization. Access rates increased substantially across income groups in a clear dose–response pattern: only 35.5% of low-income patients had accessed PC services compared with 56.3% of middle-income and 76.1% of high-income patients. The absolute difference between the highest- and lowest-income groups was 40.6 percentage points, a striking and clinically meaningful disparity.

Chi-square analysis confirmed statistical significance (χ^2^ = 21.76, *p* < 0.001; Cramér’s V = 0.33). Importantly, the linear-by-linear association test confirmed a highly significant dose–response relationship (*p* < 0.001), indicating progressively higher access with each income level increment. This dose–response pattern demonstrates that access disparities reflect systematic economic stratification rather than arbitrary clustering.

Interpretation: The magnitude of access disparities substantially exceeds awareness disparities (40.6 vs. 30 percentage points), suggesting that economic barriers operate partly through awareness gaps but also through structural mechanisms. Even patients aware of PC services face material barriers to access, such as transportation costs, lost work income, and out-of-pocket expenses, that restrict utilization to economically advantaged populations.

### 3.4. Multivariable Predictors of Palliative Care Access

Table 4 presents the results of the multivariable logistic regression analysis examining income as an independent predictor of palliative care (PC) access, adjusting for age, gender, education, geographic region, and PC service utilization.

After adjustment for all covariates, high income (>10,001 SAR) remained a statistically significant predictor of PC access (OR = 3.32; 95% CI: 1.83–6.02; *p* < 0.001).

Among the covariates, age ≥51 years was also significantly associated with higher odds of access (OR = 2.85; 95% CI: 1.61–5.01; *p* < 0.001), as was female gender (OR = 1.46; 95% CI: 1.08–2.31; *p* = 0.013).

In contrast, university education was not significantly associated with PC access (OR = 1.38; 95% CI: 0.79–2.41; *p* = 0.265). Geographic region was also not a significant predictor in the adjusted model (OR = 1.33; 95% CI: 0.91–2.45; *p* = 0.168).

PC service utilization was independently associated with access (OR = 1.51; 95% CI: 1.06–2.15; *p* = 0.022).

The overall model was statistically significant (χ^2^ = 48.67, *p* < 0.001) and explained a moderate proportion of variance in PC access (Nagelkerke R^2^ = 0.38).

Assessment of Model Assumptions: Prior to interpretation, logistic regression assumptions were systematically evaluated. Multicollinearity assessment using variance inflation factors (VIF) revealed all values < 3.0 except those for income–education (VIF = 4.8) and age–education (VIF = 3.2), indicating acceptable collinearity levels. The Hosmer–Lemeshow goodness-of-fit test confirmed adequate model calibration (χ^2^ = 4.32, *p* = 0.368). Receiver operating characteristic (ROC) curve analysis demonstrated good discriminative ability (AUC = 0.76, 95% CI: 0.69–0.83). Linearity in the logit assumption was assessed and confirmed; addition of polynomial age and education terms did not significantly improve model fit, supporting appropriate model specification.

### 3.5. Multivariable Predictors of Palliative Care Awareness

Table 5 presents the results of the multivariable logistic regression analysis examining predictors of palliative care (PC) awareness. The table was revised to improve clarity and reporting consistency: the unstandardized coefficient is labeled B (SE), PC service utilization is included as a covariate, and reference categories are specified in the footnote.

Higher income and older age were the strongest independent predictors of PC awareness. Individuals with income >10,001 SAR had nearly threefold higher odds of awareness, while those aged ≥51 years had more than double the odds. Female gender and university education were also significant predictors, though with smaller effect sizes. PC service utilization showed a modest but significant association with awareness, suggesting that experiential exposure contributes to knowledge.

Compared with access models, the income effect on awareness was slightly attenuated, indicating that socioeconomic disparities in access are driven both by differences in awareness and by structural barriers beyond awareness. Education was associated with awareness but not access, suggesting that knowledge alone may be insufficient to overcome resource-related constraints.

### 3.6. Geographic Modification of Income Effects: Stratified Analysis

Recognizing that service availability varies geographically, stratified analyses examined whether income effects on access varied between Central region (where services are concentrated) and peripheral regions (Eastern/Western combined, where services are less available).

Table 6 presents access rates by income stratum within each geographic region. Notably, income disparities were attenuated in the Central region but pronounced in peripheral regions, suggesting that geographic service availability modifies the effect of economic status on access.

In the Central region, characterized as an urban and service-rich area, income gradients in palliative care access were present but appeared substantially diminished when compared with the patterns observed in the overall sample. Specifically, access rates within this region demonstrated a progressive increase across income strata, with 45.5% of low-income patients, 62.5% of middle-income patients, and 78.9% of high-income patients reporting service utilization. This pattern suggests that while economic disparities persist even in areas with concentrated services, the baseline access for lower-income residents in these urban centers is notably higher than that of their counterparts in peripheral regions.

In contrast to the Central region, peripheral regions, characterized as rural or suburban areas with sparse service availability, demonstrated more pronounced income gradients in palliative care access. Within these underserved areas, the disparity between income groups was notably wider, with only 30.0% of low-income patients reporting access to services compared to 51.3% of middle-income patients and 72.4% of high-income patients. This 42.4 percentage-point gap between the highest- and lowest-income groups in peripheral regions substantially exceeds the disparities observed in the Central region, indicating that geographic service scarcity acts as a catalyst that amplifies existing economic barriers. These results suggest that when palliative care services are limited by distance and availability, individual financial resources become a more stringent gatekeeper for service utilization.

Interaction Analysis: The income × region interaction term approached statistical significance (*p* = 0.072), providing suggestive evidence that income disparities are more severe in peripheral regions. While not meeting conventional *p* < 0.05 threshold, this pattern carries important policy implications. The attenuated pattern (though *p* = 0.072) suggests that where services are readily available (Central region), even lower-income patients can access care at moderate rates; conversely, where services are scarce, income becomes a more stringent gatekeeper, with only higher-income populations achieving meaningful access.

## 4. Discussion

### 4.1. Magnitude and Pattern of Economic Disparities

This study documents substantial, systematic economic disparities in PC access among cancer patients in Saudi Arabia, with a striking 40.6 percentage-point gap between the highest- and lowest-income groups. This magnitude exceeds that observed for other sociodemographic factors in the multivariable model, demonstrating that economic resources constitute a fundamental determinant of PC utilization, potentially superseding clinical need or patient preference as the primary access gatekeeper.

The income gradient’s consistency across both awareness and access, combined with the dose–response pattern indicating progressive increases with income, demonstrates that these disparities reflect systematic economic stratification rather than artifact or confounding. The multivariable analysis’s finding that high income independently predicted access (OR = 3.32) even after controlling for age, gender, education, and region confirms that economic barriers operate through mechanisms distinct from other sociodemographic factors.

### 4.2. Mechanisms Underlying Income-Related Disparities

The observed income disparities likely operate through multiple specific mechanisms:

Transportation and Geographic Access Barriers: Specialized PC services concentrate in tertiary urban centers. Lower-income patients may lack private vehicle access, face substantial costs for public transit or taxi services, and lack financial resources for accommodation if travel requires overnight stays [16,30]. These barriers are particularly acute for peripheral-region patients, as reflected in the geographic stratification findings.

Opportunity Costs and Work-Related Barriers: Accessing PC frequently requires multiple appointments and consultations, necessitating time away from employment [31,32]. Lower-income individuals, particularly hourly wage workers lacking employment protections, face greater income loss and job security risks from missed work [32,33]. This opportunity cost effectively prices lower-income patients out of service access.

Out-of-Pocket Expenses: While Saudi Arabia’s public healthcare system provides substantial coverage, out-of-pocket expenses for medications, specialized consultations, and services not fully covered can accumulate substantially for chronic illnesses like cancer [10]. Lower-income households face tighter budget constraints, potentially necessitating prioritization of survival-level needs over supportive care.

Private Healthcare Access: Higher-income patients can afford private PC services with shorter wait times, greater appointment flexibility, and expanded service availability [34,35]. This creates a two-tier system wherein wealthier patients access comprehensive PC through private facilities while lower-income patients rely on public systems with longer waits and limited availability.

Information and Instrumental Resources: Beyond the awareness gaps documented in this study, higher-income patients likely possess superior access to instrumental resources, reliable transportation, flexible schedules, family support, enabling translation of awareness into actual service utilization.

Insurance Status as an Economic Mechanism: The significant association between income and insurance type reveals that private insurance, more prevalent among high-income patients, may facilitate access through reduced administrative barriers, shorter wait times, and broader provider networks. However, the multicollinearity between income and insurance (noted in the statistical methods) prevented simultaneous inclusion in the multivariable model. The primacy of income over insurance in determining access suggests that even with insurance coverage, out-of-pocket costs, transportation, and opportunity costs remain critical barriers for lower-income patients, many of whom hold government insurance with broader coverage gaps.

### 4.3. The Awareness Gradient: Knowledge as a Mediator

The income gradient in awareness (41.9% to 71.9% across income strata) suggests that knowledge differences partly explain access disparities, consistent with findings from the accompanying awareness-mediation study [36]. However, the substantially larger access gradient (35.5% to 76.1%) compared to the awareness gradient indicates that awareness differences account for only a portion of economic disparities. This pattern implies that:

1. Lower-income patients’ reduced awareness contributes meaningfully to reduced access;
2. However, structural and economic barriers operate independently of awareness, such that even economically disadvantaged patients who achieve awareness may remain unable to access services due to material barriers.

This finding carries important intervention implications: awareness-building initiatives, while valuable, must be complemented by structural interventions directly addressing economic barriers. Educational campaigns reaching lower-income populations will have limited impact if financial obstacles prevent service utilization despite knowledge.

### 4.4. Geographic Modification of Income Effects

The stratified analysis revealing that income disparities are more pronounced in peripheral regions (approaching *p* = 0.072 for interaction) illuminates the intersection of economic and geographic disadvantage. Central regions, where PC services concentrate, achieve higher access rates across all income groups, including lower-income populations (45.5%). This pattern suggests that service availability partially compensates for economic disadvantage through reducing travel burden and improving convenience [37].

Conversely, peripheral regions demonstrate that when services are scarce, economic resources become a more stringent gatekeeper. Lower-income peripheral residents face compounded disadvantages: greater travel distances requiring private transportation they may not afford, longer wait times necessitating time away from employment, and reduced service variety requiring potential travel to multiple sites [38]. These intersecting barriers reduce peripheral lower-income access to 30%, below even the already-constrained 35.5% observed in low-income populations overall.

This geographic pattern has important policy implications: geographic service expansion may represent a particularly efficient strategy for reducing economic disparities, as it addresses barriers affecting all patients but particularly impacting lower-income populations in underserved regions.

### 4.5. Comparison with International Evidence

These findings align with and extend international evidence documenting socioeconomic gradients in PC access. Nelson et al. [39] systematic review identified income and insurance status as consistent predictors of PC utilization across diverse healthcare systems. Similarly, Sítima et al. [40] reported that financial barriers constituted primary obstacles to equitable PC access in multiple countries.

However, the magnitude of disparities in this study may exceed that observed in some Western healthcare systems with more robust safety nets. The OR = 3.32 for high-income patients indicates approximately threefold increased access odds compared with lower-income groups, a substantial disparity suggesting that economic barriers may be particularly pronounced in healthcare systems with developing PC infrastructure and less comprehensive insurance coverage for supportive services.

Importantly, Saudi Arabia’s public healthcare system provides a foundation upon which equitable access could be built more readily than in fully privatized systems [41]. The findings underscore that, despite public coverage, policy measures ensuring that nominally available services are truly accessible across income strata remain essential. Public coverage alone does not guarantee equity without additional provisions addressing the structural barriers this study documents.

### 4.6. Implications for Healthcare Policy and System Transformation

These findings carry substantial implications for Saudi Arabian healthcare policy within the Vision 2030 transformation framework [8,9]:

1. Geographic Expansion of Services

The geographic modification of income effects observed in this study suggests that expanding services to peripheral regions would substantially reduce economic disparities by decreasing the travel burden and improving accessibility for all populations, particularly lower-income patients. To achieve this, several integrated strategies should be implemented, beginning with the decentralization of palliative care (PC) services to establish specialized capacity within regional hospitals and community health centers. This structural shift can be further supported by the adoption of telemedicine and remote consultation technologies, which effectively extend specialist expertise beyond centralized tertiary centers to reach underserved areas. Additionally, mobile outreach programs can play a vital role by bringing PC assessments and direct interventions to patients in their own communities. Finally, strengthening primary care integration is essential, as it enables community-based providers to deliver core PC elements, such as symptom management, psychosocial support, and advance care planning, ensuring that high-quality care is accessible regardless of a patient’s geographic or economic standing.

2. Financial Support Programs

Directly addressing out-of-pocket cost barriers, financial support programs should be implemented through several targeted mechanisms. First, healthcare systems should subsidize or eliminate transportation costs for palliative care (PC) appointments, utilizing voucher systems or the direct provision of transportation services to alleviate the financial burden on low-income patients. Furthermore, it is essential to expand insurance coverage to ensure comprehensive public reimbursement for all PC-related medications, specialized consultations, and supportive services. Policymakers should also consider establishing income-based copayment structures that scale patient contributions according to their individual ability to pay, thereby preventing financial toxicity. Finally, the implementation of robust workplace protections is necessary to ensure that time required for medical appointments does not jeopardize a patient’s employment status or lead to a significant loss of income, particularly for those in the hourly wage workforce.

3. Patient Navigation Programs

Patient navigation, which involves systematically assisting patients in overcoming logistical, financial, and informational barriers to service access, has demonstrated significant effectiveness in diverse healthcare contexts. To implement this successfully, navigation programs should first focus on identifying economically disadvantaged patients who require enhanced levels of support. Once identified, navigators must actively assist these individuals in accessing available financial resources, including insurance programs, transportation assistance, and various charitable supports. Furthermore, providing comprehensive logistical support is essential to help patients schedule appointments, arrange necessary transportation, and navigate complex bureaucratic obstacles that often impede care. Finally, navigators should serve as vital cultural brokers, helping patients better understand healthcare systems while simultaneously advocating within those systems to ensure that specific patient needs are prioritized and met.

4. Equity Monitoring and Accountability

Quality monitoring systems must be designed to systematically track disparities in palliative care (PC) access to ensure long-term accountability and transparency within the healthcare system. This process begins with the routine measurement of service access, explicitly stratified by income levels, educational attainment, and geographic regions. To foster a culture of accountability, these equity metrics should be included in public reporting, which allows stakeholders to monitor progress and identify areas requiring further intervention. Furthermore, it is essential to evaluate the effectiveness of any implemented policies by comparing access rates before and after their introduction, ensuring that resources are achieving their intended goals. Finally, ongoing data analysis and qualitative research involving underserved populations are necessary to identify any persistent or emerging barriers, thereby enabling the healthcare system to remain responsive to the needs of the most marginalized groups.

Without systematic equity measurement and public accountability, expanding service systems risk further entrenching disparities by concentrating enhanced services in geographic areas and income strata most capable of accessing centralized facilities.

### 4.7. Implications for Nursing Practice and Advocacy

Nurses can address economic barriers to PC access through multiple practice-level and systems-level strategies:

1. Patient-Level Assessment and Advocacy

To effectively address the economic disparities identified in this study, several patient-level strategies must be integrated into nursing practice. First, systematic assessment of economic barriers should be formally incorporated into oncology nursing assessments, enabling the early identification of patients facing financial constraints, transportation limitations, or work-related obstacles to care. By maintaining a comprehensive knowledge of available support programs, including insurance benefits, transportation assistance, charitable organizations, and workplace supports, nurses are strategically positioned to connect patients with the necessary financial resources to overcome these material barriers. Furthermore, persistent advocacy for individual patients remains crucial to ensuring that economic constraints do not preclude service access. This advocacy involves dedicated referral efforts, meticulous documentation of social barriers, and the necessary escalation of concerns within healthcare systems to prioritize equitable care for underserved populations.

2. Program-Level Initiatives

At the program level, oncology nursing leadership should drive the development and implementation of structured patient navigation programs. Nurses’ deep clinical knowledge and established patient relationships position them as ideal leaders for navigation initiatives that specifically address the logistical and financial barriers identified in this study. Furthermore, the integration of palliative care into early cancer care is essential, as it may reduce overall access barriers by establishing awareness and rapport earlier in the illness trajectory, a time when patients often maintain greater physical and financial capacity for engagement. To support this, patient education must be culturally appropriate and specifically address economic realities by explaining how to access financial support, navigate transportation hurdles, and connect with community resources. Rather than assuming patients can overcome material barriers independently, these educational efforts should provide practical, accessible guidance tailored to the socioeconomic challenges of the population.

3. Advocacy and Policy Engagement

Professional advocacy through nursing organizations is essential for advancing policies that directly address the economic barriers identified in this research, including geographic service expansion, the establishment of financial support programs, and the nationwide implementation of patient navigation and equity monitoring systems. By presenting evidence-based findings to policymakers, nurses can build a compelling case for equity-focused investments, framing them as both an ethical imperative and a highly efficient healthcare strategy. This approach emphasizes that existing gaps in service utilization represent underutilized capacity and that closing these gaps through earlier intervention can effectively prevent costly complications. Ultimately, through professional engagement in policy discourse, nurses can ensure that healthcare transformation efforts achieve an equitable distribution of services across all socioeconomic groups.

### 4.8. Strengths

This study demonstrates several significant methodological strengths that enhance the validity and relevance of its findings. A primary strength lies in the explicit income stratification, which enabled a systematic examination of dose–response relationships and economic gradients across the participant sample. Furthermore, the use of validated measurement instruments, rigorously adapted and translated for the Saudi context, ensures the cultural and linguistic appropriateness of the data collected. The application of multivariable analysis was also critical, as it allowed for the isolation of income effects from potential confounding factors by controlling for various correlated sociodemographic variables. Additionally, the study’s stratified geographic analysis provided a sophisticated understanding of how regional service availability modifies the impact of income on access. By recruiting participants across diverse healthcare settings, the research effectively reduced the selection bias inherent in single-site studies. Finally, the clear policy relevance of this research, which is directly aligned with the Vision 2030 healthcare transformation priorities, ensures that these findings provide actionable insights for national health system development.

### 4.9. Limitations

Several limitations warrant acknowledgment to contextualize these findings. First, the cross sectional design precludes causal inferences and prevents the examination of temporal access patterns across illness trajectories; the possibility of reverse causation also cannot be entirely excluded. Second, reliance on self-reported income may introduce social desirability bias, while the use of broad income categories might mask more nuanced income–outcome relationships. Third, important confounders such as insurance type, comorbidities, and specific cultural beliefs were not fully examined in the multivariable model due to multicollinearity concerns with income; this represents a substantive limitation requiring discussion. Fourth, the study was restricted to Riyadh, the findings may not be fully generalizable to peripheral regions with less developed healthcare infrastructure. Fifth, the analysis may also be influenced by unmeasured confounders, such as insurance type, comorbidities, and specific cultural beliefs. Finally, the use of stratified purposeful sampling rather than random sampling may have introduced selection bias toward patients already engaged with the healthcare system, potentially underestimating the true magnitude of disparities among the most marginalized populations.

### 4.10. Future Research Directions

Future investigations should address the limitations of the current study through several strategic research pathways. Longitudinal designs are needed to track cancer patients prospectively across their illness trajectories, which would allow for a more nuanced examination of how shifting economic circumstances influence the timing of palliative care (PC) awareness and access. Complementing this, qualitative research exploring the perspectives of patients and families could provide deeper insights into the lived experiences of economic barriers and the diverse strategies families employ to overcome them. To enhance generalizability, future studies should expand to other regions of Saudi Arabia to identify region-specific factors that may modify the relationship between income and access. Furthermore, intervention studies are essential to evaluate the effectiveness of specific strategies, such as geographic expansion, financial support programs, and patient navigation, in successfully reducing economic disparities. These efforts should be supported by cost-effectiveness analyses to demonstrate the broader economic benefits of equity-focused investments, including reduced emergency department utilization and improved workforce productivity. Finally, mechanistic research is required to more specifically identify which economic barriers, such as transportation costs versus opportunity costs, most significantly constrain access, thereby enabling the precise prioritization of interventions.

### 4.11. Practical Implications and Recommendations

Based on the findings of this study, several actionable recommendations are proposed for healthcare systems, policymakers, and nursing leadership. For healthcare systems and policy development, the priority should be the geographic expansion of PC services to underserved regions through decentralization, the scaling of telemedicine, and deeper integration with primary care providers. These structural changes must be paired with comprehensive financial support programs that include transportation subsidies, expanded insurance coverage for PC-related services, and the establishment of workplace protections for patients and their caregivers. Additionally, the implementation of dedicated patient navigation programs and routine equity monitoring systems is vital to ensure transparency and accountability in tracking access disparities. Integrating PC earlier into the cancer diagnosis and treatment planning phase is also recommended to establish awareness before economic constraints become insurmountable barriers.

Nursing leadership and practice also play a critical role in promoting equity. Systematic economic barrier assessments should be integrated into routine oncology nursing practice, requiring nurses to develop the specific knowledge and skills necessary to connect patients with available financial resources and support programs. Nurses must remain persistent in their advocacy for individual patients facing economic hurdles and take the lead in developing nursing-led patient navigation initiatives. Furthermore, engaging in professional advocacy for policy changes and incorporating discussions of economic barriers into nursing education and continuing professional development will ensure that the nursing workforce is prepared to address the socioeconomic determinants of health effectively.

## 5. Conclusions

This study identifies significant income-based disparities in palliative care (PC) access among Saudi Arabian cancer patients, driven by both awareness gaps and structural economic barriers such as transportation and out-of-pocket costs. These inequities are notably amplified in peripheral regions, where geographic service scarcity compounds financial vulnerability. Addressing these disparities requires a multifaceted strategy, including financial support programs, geographic service expansion, patient navigation, and robust equity monitoring systems. Nurses, as frontline providers, are essential catalysts for change through systematic socioeconomic assessments, patient advocacy, and leadership in navigating healthcare barriers. Within the Vision 2030 framework, prioritizing economic equity in PC access is both an ethical imperative and a systemic necessity to ensure the equitable distribution of healthcare resources across all socioeconomic strata.

## Figures and Tables

**Table 1:** Demographic Characteristics by Income Group.

Variable	Low Income (<5000 SAR) *n* = 62	Middle Income (5001–10,000 SAR) *n* = 71	High Income (>10,001 SAR) *n* = 67	*p* Value
Age, Mean (SD)	43.9 (9.8)	48.1 (9.4)	52.8 (8.7)	<0.001
Female, *n* (%)	33 (53.2)	38 (53.5)	30 (44.8)	0.337
University Education, *n* (%)	16 (25.8)	33 (46.5)	49 (73.1)	<0.001
Central Region, *n* (%)	22 (35.5)	32 (45.1)	38 (56.7)	0.016
Peripheral Region, *n* (%)	40 (64.5)	39 (54.9)	29 (43.3)	—
Government Insurance, *n* (%)	38 (61.3)	35 (49.3)	15 (22.4)	<0.001
Private Insurance, *n* (%)	24 (38.7)	36 (50.7)	45 (67.2)	—
PC Services Accessed, *n* (%)	22 (35.5)	40 (56.3)	51 (76.1)	<0.001
PC Services Not Accessed, *n* (%)	40 (64.5)	31 (43.7)	16 (23.9)	—

**Table 2:** Palliative Care Awareness by Income Stratum.

Income Group	Low Awareness *n* (%)	High Awareness *n* (%)	Total	χ^2^ (*p* Value)
Low (<5000 SAR)	36 (58.1)	26 (41.9)	62	17.89 (0.001)
Middle (5001–10,000 SAR)	32 (45.1)	39 (54.9)	71	-
High (>10,001 SAR)	19 (28.1)	48 (71.9)	67	-
Total	87 (43.5)	113 (56.5)	200	-

Linear by linear association (dose response): *p* < 0.001.

**Table 3:** Palliative Care Access by Income Stratum.

Income Group	Accessed PC *n* (%)	Not Accessed PC *n* (%)	Total	χ^2^ (*p* Value)
Low (<5000 SAR)	22 (35.5)	40 (64.5)	62	21.76 (<0.001)
Middle (5001–10,000 SAR)	40 (56.3)	31 (43.7)	71	-
High (>10,001 SAR)	51 (76.1)	16 (23.9)	67	-
Total	113 (56.5)	87 (43.5)	200	-

Linear by linear association (dose response): *p* < 0.001.

**Table 4:** Multivariable Logistic Regression: Predictors of Palliative Care Access.

Predictor	B (SE) ᵃ	OR (95% CI)	*p*-Value
High Income (>10,001 SAR)	1.20 (0.33)	3.32 (1.83–6.02)	<0.001
Age ≥ 51 years	1.05 (0.28)	2.85 (1.61–5.01)	<0.001
Female Gender	0.38 (0.15)	1.46 (1.08–2.31)	0.013
University Education	0.32 (0.29)	1.38 (0.79–2.41)	0.265
Central Region	0.29 (0.22)	1.33 (0.91–2.45)	0.168
PC Service Utilization (accessed = yes)	0.41 (0.18)	1.51 (1.06–2.15)	0.022
Model χ^2^	48.67	—	<0.001
Nagelkerke R^2^	0.38	—	—

Note: ᵃ B = unstandardized coefficient; SE = standard error. Reference categories: income (low income, <5000 SAR), age (<51 years), gender (male), education (secondary or less), region (peripheral), and PC service utilization (not accessed).

**Table 5:** Multivariable Logistic Regression: Predictors of Palliative Care Awareness.

Predictor	B (SE) ᵃ	OR (95% CI)	*p*-Value
High Income (>10,001 SAR)	1.08 (0.32)	2.95 (1.57–5.52)	<0.001
Age ≥ 51 years	0.87 (0.26)	2.39 (1.44–3.97)	<0.001
Female Gender	0.31 (0.14)	1.36 (1.03–1.79)	0.031
University Education	0.62 (0.27)	1.86 (1.09–3.16)	0.022
Central Region	0.28 (0.21)	1.32 (0.88–1.98)	0.178
PC Service Utilization (accessed = yes)	0.38 (0.19)	1.46 (1.01–2.12)	0.046
Model χ^2^	42.18	—	<0.001
Nagelkerke R^2^	0.35	—	—

ᵃ B = unstandardized beta coefficient; SE = standard error. Reference categories: Income (<5000 SAR); Age (<51 years); Gender (male); Education (secondary or less); Region (peripheral); PC utilization (not accessed).

**Table 6:** Palliative Care Access by Income Stratum and Geographic Region (Stratified Analysis).

Region	Income Group	Accessed PC *n* (%)	Not Accessed PC *n* (%)	Total
Central	Low (<5000 SAR)	10 (45.5)	12 (54.5)	22
	Middle (5001–10,000 SAR)	20 (62.5)	12 (37.5)	32
	High (>10,001 SAR)	30 (78.9)	8 (21.1)	38
	Subtotal	60 (64.5)	32 (35.5)	92
Peripheral	Low (<5000 SAR)	12 (30.0)	28 (70.0)	40
	Middle (5001–10,000 SAR)	20 (51.3)	19 (48.7)	39
	High (>10,001 SAR)	21 (72.4)	8 (27.6)	29
	Subtotal	53 (56.4)	55 (58.5)	108
Interaction (Income × Region):				*p* = 0.072

## Data Availability

The original contributions presented in this study are included in the article. Further inquiries can be directed to the corresponding author.

## Abbreviations

The following abbreviations are used in this manuscript:

PC	Palliative Care
PCAAS	Palliative Care Awareness and Accessibility Scale
SAR	Audi Riyal
OR	Odds Ratio
CI	Confidence Interval
LMICs	Low- and Middle-Income Countries
IRB	Institutional Review Board
KSU	King Saud University
MOH	Ministry of Health
